# Effects of marketing claims on toddler food products on parents’ product preferences, perceptions and purchasing intentions: an online experiment

**DOI:** 10.1186/s12966-024-01603-9

**Published:** 2024-05-21

**Authors:** Helen Gwenda Dixon, Mamaru Ayenew Awoke, Maree Scully, Jennifer McCann, Jane Martin, Belinda Morley, Anthea Rhodes, Alison McAleese, Andrea Schmidtke

**Affiliations:** 1 Centre for Behavioural Research in Cancer, Cancer Council Victoria, 200 Victoria Pde., East Melbourne, VIC 3002 Australia; 2 School of Psychological Sciences, The University of Melbourne, Parkville, VIC Australia; 3 Institute for Physical Activity and Nutrition (IPAN), School of Exercise and Nutrition Sciences, Deakin University, Burwood, VIC Australia; 4 Food for Health Alliance, Melbourne, VIC Australia; 5 The Royal Children’s Hospital Melbourne, Parkville, VIC Australia; 6 Department of Paediatrics, The University of Melbourne, Parkville, VIC Australia; 7 Prevention Division, Cancer Council Victoria, Melbourne, VIC Australia

**Keywords:** Toddler foods, Marketing claims, Parents, Online experiment, Policy, Food marketing

## Abstract

**Background:**

The retail market for toddler-specific packaged foods is growing. Many of these products are ultra-processed and high in nutrients of concern for health, yet marketed in ways that may make them appear wholesome. This study aims to assess parents’ responses to claims on unhealthy, ultra-processed toddler food products and test whether removing such claims promotes more accurate product perceptions and healthier product preferences.

**Methods:**

Parents of toddlers aged 12 to < 36 months (*N* = 838) were recruited for an online experiment testing four on-pack claim conditions: control (no claim); 'contains "good" ingredient'; 'free from "bad" ingredient'; and unregulated 'child-related' claim. Participants were randomly assigned to one condition, then viewed images of toddler food products that varied in nutrition content and the claims displayed. Participants completed tasks assessing product preferences (unhealthy product displaying claim vs. a healthier option with no claim, across four food categories (banana bars, strawberry snacks, blueberry yogurt snacks and veggie snacks)), purchase intentions and product perceptions. Poisson regression (count variable) and linear regression (continuous outcomes) analyses were employed to test for mean differences by marketing claim conditions.

**Results:**

For the overall sample, brief exposure to ‘free from "bad" ingredient’ claims increased participant’s intentions to purchase unhealthy food products for their toddlers, but there was no clear evidence that ‘contains "good" ingredient’ claims and ‘child-related’ claims significantly impacted parent’s preferences, purchase intentions and perceptions of toddler foods. However, certain claims influenced particular parent subgroups. Notably, parents with three or more children chose more unhealthy products when these products displayed ‘contains "good" ingredient’ or ‘free from "bad" ingredient’ claims; the latter claims also promoted stronger purchase intentions and enhanced product perceptions among this subgroup.

**Conclusions:**

Findings indicate that ‘free from "bad" ingredient’ claims on unhealthy toddler foods are of most concern, as they boost the appeal of these products to parents. ‘Contains "good" ingredient’ claims and ‘child-related’ claims showed limited effects in this study. Considering available evidence, we recommend claims should not be permitted on child-oriented foods, as they may promote inaccurate product perceptions and unhealthy product choices by parents, that can detract from their children’s diets and health.

**Supplementary Information:**

The online version contains supplementary material available at 10.1186/s12966-024-01603-9.

## Introduction

Young children (< 3 years) need good nutrition for optimal growth and development, to establish healthy habits and reduce the risk of chronic disease later in life [1, 2]. It is recommended that from 12 months of age, toddlers (aged 12–36 months) should eat family foods, consistent with the Australian Dietary Guidelines (ADGs) [3]. Unhealthy foods of low nutritional value and high saturated fat, sugar and or salt should be restricted for toddlers. Parents play a critical role in shaping their children’s eating habits and food choices as the primary provider and influencers of their children’s diet [4, 5]. However, broader socio-ecological factors such as availability and affordability of healthy foods, the marketing and promotion of unhealthy foods, and cultural and social norms around food and eating habits can impact parents’ ability to provide nutritious foods for their children [6].

There has been steady market growth for readymade infant and toddler foods in Australia over recent decades [7–9], including a notable increase in the availability of toddler-specific foods [10]. Concerningly many of these products are ultra-processed foods (UPFs) and contain high amounts of fat, salt and/or sugar [7, 11, 12]. As per the NOVA food classification system, UPFs have little similarity to traditional food items as they are comprised of formulations of ingredients, mostly of exclusive industrial use. UPFs often contain additives, preservatives, and other chemicals and are typically high in unhealthy nutrients such as sugar, fat, and salt [13]. In the last decade, new toddler-specific products launched in Australia have contained more sugar, fat and saturated fat, but lower sodium, than in earlier years [10]. While some clear standards exist in relation to infant foods (e.g., limiting sodium), Australia’s food regulatory system allows infant and toddler-specific food products that are UPFs and particularly high in added sugars to be produced and sold [11, 14]. The food industry's increasing reliance on ultra-processed ingredients has raised concerns about the nutritional quality and health impacts of processed foods, particularly those marketed for infants and toddlers.

Many parents rely on ready-made foods for their infants and toddlers [15]. A parent poll, conducted in Melbourne, Victoria, found that almost one in two infants and toddlers consume ready-made foods at least 2–3 times weekly, with less educated and sole parents reporting more frequent consumption [15]. Two out of five parents agreed these foods must be healthy, or the government wouldn’t allow them to be sold; parents with less education were more inclined to agree that ready-made foods are healthier than food made at home [15]. These findings are particularly concerning from an equity perspective, as they indicate that more disadvantaged parents are especially reliant on ready-made foods, and more likely to perceive them as healthier than homemade foods.

In addition to concerns surrounding the composition of infant and toddler foods, the claims commonly displayed on these products also warrant scrutiny. Food Standards Australia New Zealand’s (FSANZ’s) Food Standard Code (FSC) sets out strict criteria for claims that suggest that a food or property of food, has, or may have, a health effect. For example, for a product to carry a claim that calcium enhances bone mineral density, it must contain no less than 200 mg of calcium per serving. Other nutrition content claims, that simply state the presence or absence of a property in a food (e.g., ‘contains whole grains’ or ‘free from artificial flavours’) are expressly permitted by the FSC but there are no criteria that the products need to meet – claims like these are only permitted to state that the product contains that particular property or not, and, if present, how much it contains [16]. Consequently, products may display claims about the presence or absence of certain nutrients or ingredients, irrespective of the healthfulness of their overall nutritional profile.

More general claims also commonly appear on toddler food products, which are not regulated under the FSC. These unregulated claims are varied but often focus on the source or nature of ingredients (e.g., all natural or organic), or the child consumer (e.g., perfect for little hands) [7]. Between 1996 and 2020, the number of claims per package increased for toddler foods, coupled with an increase in unregulated claims [7]. Claims appearing on these products often imply they are superior to whole foods and essential to children’s healthy growth and development, despite many having a poor nutritional profile. For example, Simmonds et al.’s cross-sectional audit conducted in August 2019 found the majority (85%) of fruit and vegetable-based infant foods displaying statements suggesting low sugar content were sweetened with fruit puree (free sugars) [17], resulting in high total sugar[18].

Consumers are known to be influenced by marketing on food packaging at the point-of-sale and during consumption [19]. The concern about claims highlighting isolated positive attributes of otherwise unhealthy products is that they produce a cognitive bias, known as the ‘health halo effect’, whereby consumers generalise from this positive attribute to their overall appraisal of a product’s healthiness, regardless of the total nutritional profile [20, 21]. A direct choice experiment found that nutrition content claims were especially influential, followed by unregulated claims, in increasing parent’s perceptions of the healthiness of UP toddler foods and milks, providing a ‘health halo’ for these products, leading the authors to call for further controls to regulate the use of such claims to facilitate informed consumer choice [10]. Previous experiments with school-aged children and their parents found nutrient content claims on child-oriented food products can tip preferences towards unhealthy food options displaying these claims [22, 23].

Concerns about the nutritional suitability and marketing of infant and toddler foods are not unique to Australia, with the World Health Organization (WHO) Europe proposing a Nutrient and promotion profile model (NPPM) to address this issue [18]. The NPPM sets requirements relating to the composition of infant and toddler food products as well as to promotional messages and packaging, including that no compositional, nutritional, health or marketing claims be permitted on these products. A 2022 audit applying WHO’s NPPM model to infant and toddler foods sold in major Australian supermarket chains found a minority (28%) met all compositional requirements, with toddler foods least likely to be compliant (18%). Notably, every product was found to carry multiple claims that would not be permitted under WHO Europe’s NPPM [24].

In addition to considering different types of claims (e.g., health, nutrient, or marketing claims), psychological theory and advertising research draws attention to specific motivational appeals that are commonly used within such claims. Claims highlighting the presence of positive product attributes (e.g., 'high in "good" ingredient') appeal to benefit-seeking motivation, whereas claims highlighting the absence of negative product attributes (e.g., 'free from "bad" ingredient') appeal to risk-avoidance motivation [21]. Research testing responses to nutrition content claims in food and drink marketing in women’s magazines found that both benefit-seeking and risk-avoidance appeals enhance the perceived healthiness of marketed products (even though the product may be generally unhealthy), and women report preferring risk avoidance appeals [21]. Multi-disciplinary theory and research from other food product domains demonstrates that consumers’ responses to health claims results from an interplay between characteristics of the claims displayed (ingredients, wording, positioning, visual aids, scientific communication) and the consumer (socio-demographic, knowledge, familiarity, attitudes) [25]. There is every reason to expect that similar factors may be at play in determining parents’ responses to claims on toddler foods.

Research is needed to improve our understanding of how common types of claims and motivational appeals that appear on unhealthy toddler foods influence parents’ and carers’ perceptions of the healthfulness of these products, and ultimately their decisions on what to purchase and feed their young children. To our knowledge, no published research has yet examined how various claim types and motivational appeals appearing on unhealthy toddler snack foods influence parent’s preferences, purchasing intentions and perceptions of product healthiness.

The present study aims to assess parents’ responses to the following archetypal, claims that appear on unhealthy, toddler snack foods: *contains ‘good’ ingredient* (ingredient-related claim emphasising benefit-seeking motivational appeal); *free from ‘bad’ ingredient* (ingredient-related claim emphasising risk-avoidance motivational appeal); *has ‘good’ child-related feature* (child-related claim emphasising benefit seeking motivational appeal), and whether removing such claims promotes more accurate product perceptions and healthier product preferences. It is hypothesised that displaying these types of claims on unhealthy toddler snack foods will enhance parent’s perceptions of the healthiness and appropriateness of these products for toddlers, as well as their preferences and purchase intentions for these products compared to when no claim is displayed. We also explore whether some claims are more influential than others (especially risk-avoidance claims [21, 26]), and whether certain demographic sub-groups of parents are more susceptible to influence by the respective claims.

## Method

### Design and procedure

**Fig. 1:**
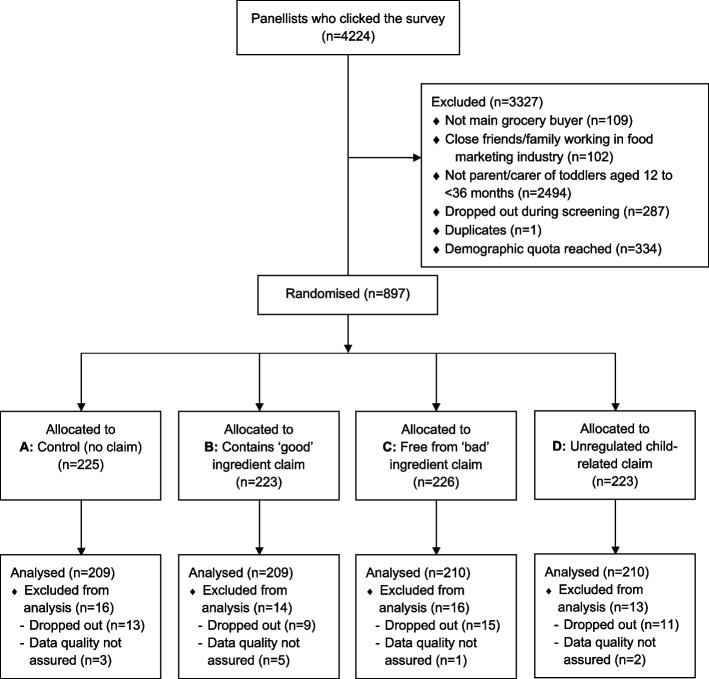
Participant flow diagram

Using a between-subjects online experimental design, parents/carers of toddler/s (aged 12 to < 36 months) who are responsible for most food purchases for their toddler/s were randomly assigned to one of four claim conditions: A, Control (no claim, simulating ban); B, Contains ‘good’ ingredient claim (e.g., made with wholegrain); C, Free from ‘bad’ ingredient claim (e.g., no additives); and D, Unregulated child-related claim (e.g., perfect for little hands) (Fig. 1). Participants viewed images of toddler food products that displayed claims reflecting their assigned condition, then completed the response measures. Upon completion, participants were debriefed on the study aims and provided with weblinks to summary information about the ADGs for Children.

### Participants

Participants were recruited through a non-probability online panel managed by Ipsos Social Research Institute, with members sent an email invitation with a link to an explanatory statement, consent form, and survey. The online consent form described the study as being about parent’s product choices for their toddler (the experimental manipulation was not revealed until the end of the survey). Screening questions at the beginning of the survey assessed eligibility (e.g., parent of toddler (12 to < 36 months)). Parents with more than one qualifying child responded in relation to their youngest toddler.

### Stimuli

**Fig. 2:**
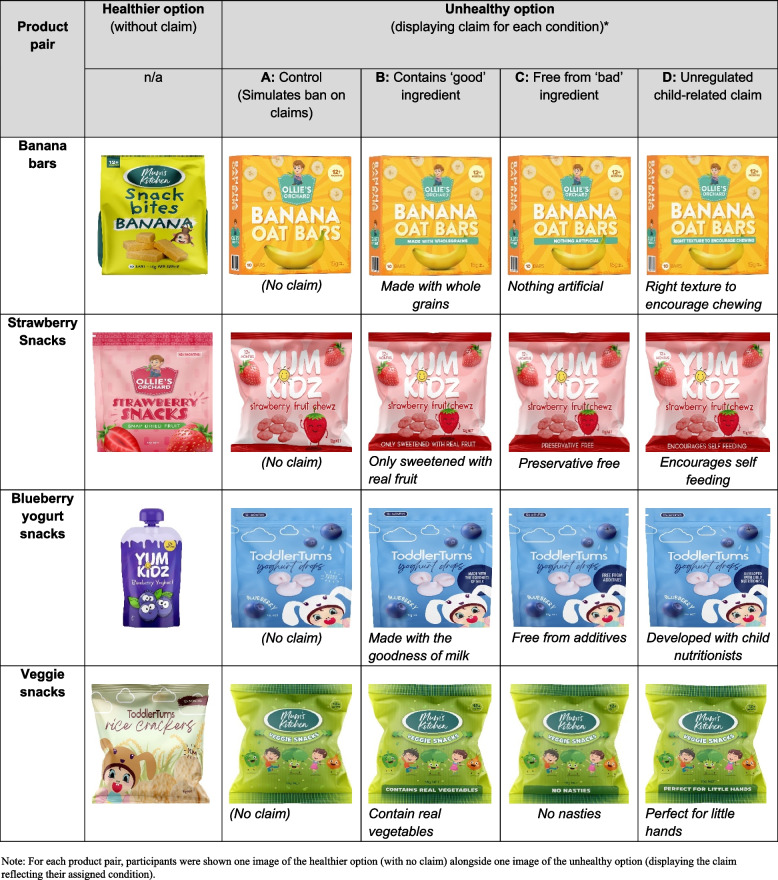
Images of mock toddler snack food  product pairs, where the healthier option displayed no claim and the unhealthy option displayed a claim representing participant's assigned condition

Full-colour digital images of eight mock toddler snack food products were created by a graphic designer. The eight product images consisted of two products (an unhealthy and a healthier option) from each of the four product categories listed in Fig. 2. The product images were created to mimic common Australian toddler snack foods, with packaging features altered to avoid known products or brand preferences. Within each product pair, the flavour (e.g., banana) was held constant to minimise potential confounding. Participants could click on each product image to view its nutrition information panel (NIP) and ingredients list. The *unhealthy products’* NIPs indicated they were high in added sugars (≥ 20g/100g) and sodium (≥ 360mg/100g) and were UP (as per the NOVA system, i.e., containing 5 or more ingredients, including substances extracted from foods and derived from further processing, and may contain food additive classes such as colours, flavours, non-sugar sweeteners, and processing aids [27]). The *healthier products’* NIPs indicated they were lower in these nutrients of concern (added sugars < 20g/100g; sodium < 120mg/100g) and were not UP. The experimental manipulation was operationalised by displaying claims reflecting each condition on the front of the package of the unhealthy product in each pair. Here, we tested nutrition content claims as well as claims that are not regulated under the FSC. The unregulated claims tested relate to the child rather than any property in the food (e.g., perfect for little hands). Figure 2 shows the product pairs and the respective front-of pack marketing claims tested in each condition. For each product pair, participants viewed the image of the unhealthy product with a claim reflecting their assigned condition (A, B, C or D) alongside the healthier option with no claim.

### Outcome measures

#### Product preferences

To measure parents’ product preferences, participants were presented with the four product pairs in random order and asked to choose which snack they would be most likely to purchase for their toddler. Each pair consisted of an unhealthy and a healthier option, with claims reflecting the respective conditions displayed on the unhealthy option (Fig. 2). The order of presentation of the two products on screen for each pair (e.g., left vs. right) was also randomised. A count variable (possible range: 0–4) was created to indicate the number of times an unhealthy option was chosen across the four product pairs.

#### Product purchase intentions

Participants were shown the unhealthy option from each product pair on its own and asked how likely they would be to buy that product for their toddler if it were available where they normally shop. Responses were recorded on a 7-point scale ranging from ‘very unlikely’ to ‘very likely’. A mean score was computed by averaging the four product ratings.

#### Product perceptions

Participants’ perceptions of each unhealthy product option, including their health and nutritional value, were assessed. To gauge general perceptions, participants were asked to rate their level of agreement (ranging from 1 = ‘strongly disagree’ to 7 = ‘strongly agree’) that the food product is ‘good for toddlers’ health, growth, and development’, ‘suitable as part of a healthy diet for toddlers’, and ‘an everyday food for toddlers’. Participants also rated how healthy they considered each unhealthy option on a 7-point scale ranging from ‘not healthy at all’ to ‘very healthy’. A mean agreement score was created by averaging the four product ratings.

### Demographics

Participants were asked their gender, age, highest level of educational attainment, parental status (sole/co-parent), language, residential postal code, and number of children in the household. Additionally, the frequency with which their toddler eats ready-made toddler foods in a typical week was recorded. Area-based socio-economic status (SES) was estimated according to the Australian Bureau of Statistics' Index of Relative Socio-Economic Disadvantage using participant's residential postal code.

### Statistical analysis

Data were analysed using Stata/MP V.16.1 (Stata Corp) [28]. Descriptive statistics, including chi-square tests were used to test differences in proportions of participants’ demographic characteristics across marketing claim conditions. Poisson regression (count variable: number of times choosing unhealthy products) and linear regression (continuous outcomes: product purchase intention, overall product perceptions and perceived healthiness of the product) analyses tested for mean differences by marketing claim condition, with the no claim (control) condition specified as the reference category.

Exploratory analyses tested for interactions between marketing claim condition and participant’s socio-demographic characteristics listed in Table 1 (potential moderators) on each outcome measure. To examine interaction effects, each regression model was rerun with indicators for condition, potential moderator (socio-demographic characteristics), and interaction terms between condition and levels of the moderator. Since detecting interactions often requires a larger sample size compared to detecting a main effect, interactions are particularly difficult to detect and prone to Type II error; therefore, an interaction effect was considered statistically significant if the *p*-value for the interaction term was < 0.20 [29, 30]. Several recent studies have also used a similar approach, employing a *p*-value threshold of < 0.20 for testing interactions [31–33]. Where a significant interaction was found overall, separate analyses were conducted for each level of the moderator variable and *p* < 0.05 was accepted.

**Table 1:** Socio-demographic characteristics of the sample

Characteristic	Total (*N* = 838)	Control (no claim) (*n* = 209)	Contains ‘good’ ingredient (*n* = 209)	Free from ‘bad’ ingredient (*n* = 210)	Unregulated child-related claim (*n* = 210)
**Gender**^**a**^					
Male	232 (27.7)	49 (23.4)	68 (32.5)	54 (25.7)	61 (29.1)
Female	602 (71.8)	160 (76.6)	139 (66.5)	154 (73.3)	149 (71.0)
Other	4 (0.50)	0 (0)	2 (0.96)	2 (0.95)	0 (0)
**Age**					
18–29 years	225 (26.9)	59 (28.2)	58 (27.8)	50 (23.8)	58 (27.6)
30–39 years	350 (41.8)	84 (40.2)	88 (42.1)	89 (42.4)	89 (42.4)
40 + years	263 (31.4)	66 (31.6)	63 (30.1)	71 (33.8)	63 (30.0)
**Highest level of education**					
Did not complete tertiary	427 (51.0)	107 (51.2)	108 (51.7)	105 (50.0)	107 (51.0)
Tertiary or higher	411 (49.1)	102 (48.8)	101 (48.3)	105 (50.0)	103 (49.1)
**SEIFA**^**b**^					
Low (Q1 & 2)	285 (34.0)	68 (32.5)	78 (37.3)	79 (37.6)	60 (28.6)
Medium (Q3 & 4)	399 (47.6)	101 (48.3)	93 (44.5)	92 (43.8)	113 (53.8)
High (Q5)	154 (18.4)	40 (19.1)	38 (18.2)	39 (18.6)	37 (17.6)
**Frequency that toddler eats ready-made toddler foods**					
≤ 1 day/week	203 (24.2)	58 (27.8)	45 (21.5)	45 (21.4)	55 (26.2)
2–3 days/week	250 (29.8)	59 (28.2)	65 (31.1)	69 (32.9)	57 (27.1)
4–6 days/week	185 (22.1)	44 (21.0)	49 (23.4)	48 (22.9)	44 (21.0)
Every day of the week	200 (23.9)	48 (23.0)	50 (23.9)	48 (22.9)	54 (25.7)
**Language other than English**					
Yes	152 (18.1)	36 (17.2)	35 (16.8)	39 (18.6)	42 (20.0)
No	686 (81.9)	173 (82.8)	174 (83.3)	171 (81.4)	168 (80.0)
**Number of children**					
One	315 (37.6)	81 (38.8)	88 (42.1)	68 (32.4)	78 (37.1)
Two	344 (41.0)	84 (40.2)	80 (38.3)	90 (42.9)	90 (42.9)
Three or more	179 (21.4)	44 (21.1)	41 (19.6)	42 (20.0)	42 (20.0)
**Sole (single) parent**					
Yes	250 (29.8)	64 (30.6)	55 (26.3)	64 (30.5)	67 (31.9)
No	588 (70.2)	145 (69.4)	154 (73.7)	146 (69.5)	143 (68.1)

Numbers in parentheses are percentages. Percentages may not sum to 100% due to rounding. Exploratory analyses were performed comparing male vs. female by excluding the ‘other’ category to observe gender specific effects, and there were no changes in the pattern of results

^a^For multivariable analyses, participants who reported their gender as ‘male’ or ‘other’ were grouped together to retain sufficient statistical power

^b^SEIFA, socio-economic index for areas, which was determined according to the Australian Bureau of Statistics Index of Relative Socio-Economic Disadvantage ranking for Australia using participant’s residential postcode. This index ranks areas on a continuum of disadvantage (from most disadvantaged to least disadvantaged) taking into consideration characteristics that may enhance or reduce socio-economic conditions of the area

## Results

### Sample characteristics

A total of 838 parent/carers of toddlers participated (Fig. 1). The sample’s socio-demographic profile is summarised in Table 1, with characteristics balanced across claim conditions. Most participants were female (72%), and 42% were aged 30–39 years. Nearly half of the participants (49%) had completed tertiary education, 30% reported that their toddlers consumed readymade foods 2–3 days per week, and the majority (70%) were co-parents. Around one in five spoke a language other than English (18%) or had three or more children (21%).

### Purchase preference

**Fig. 3:**
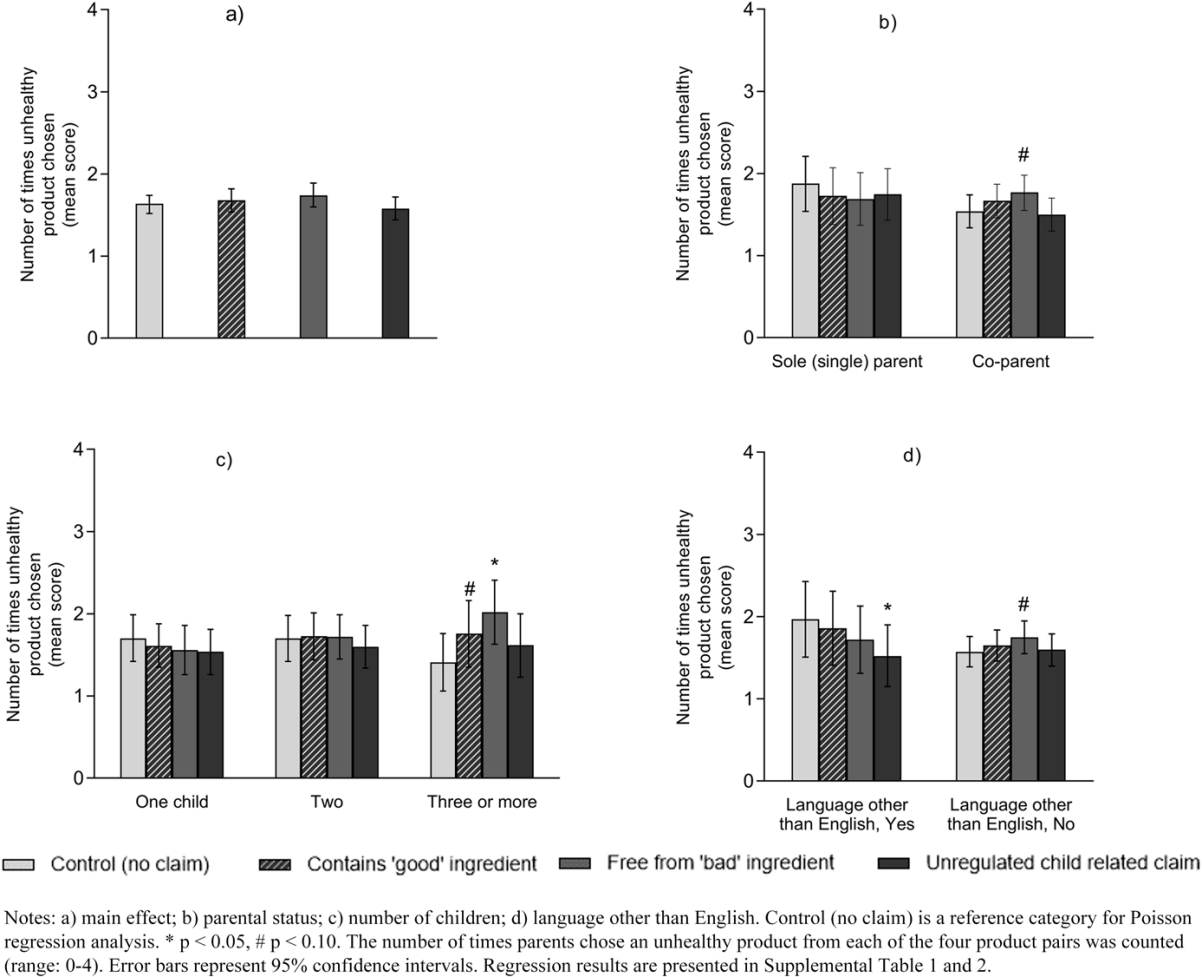
Mean score of participants' unhealthy product choices from each of the four product pairs (*N* = 838)

For the overall sample, participants’ preferences for purchasing unhealthy products were not affected by claim conditions compared to control (no claim) (Fig. 3 and Supplemental Table 1). However, for this outcome, there were interactions between claim condition and parental status (χ2 (3) = 5.12, *p* = 0.1633), language (χ2 (3) = 5.96, *p* = 0.1133), and number of children (χ2 (6) = 9.8, *p* = 0.1332) respectively. Despite the weak strength of interaction effects, co-parents (M: 1.77 vs. 1.54, *β* = 0.14, 95%CI -0.00, 0.28, *p* = 0.053), only English speakers (M: 1.75 vs. 1.57, *β* = 0.11, 95%CI -0.02, 0.23, *p* = 0.099) and those with three or more children (M: 2.02 vs. 1.41,* β* = 0.36, 95%CI 0.14, 0.58, *p* = 0.001) who were exposed to ‘free from "bad" ingredient’ claims chose unhealthy toddler products on more occasions compared to control (Fig. 3). Whereas parents who speak a language other than English (M: 1.52 vs. 1.97, *β* = -0.26 95%CI -0.47, -0.05, *p* = 0.015) who were exposed to unregulated ‘child-related’ claims chose unhealthy toddler products on fewer occasions compared to such parents in the control condition (Fig. 3d and Supplemental Table 2). Additionally, parents with three or more children (M: 1.76 vs. 1.41,* β* = 0.22, 95%CI -0.03, 0.47, *p* = 0.081) exposed to ‘contains "good" ingredient’ claims chose unhealthy toddler products a moderately higher number of times compared to those in the control condition (Fig. 3c and Supplemental Table 2).

### Purchase intentions

**Fig. 4:**
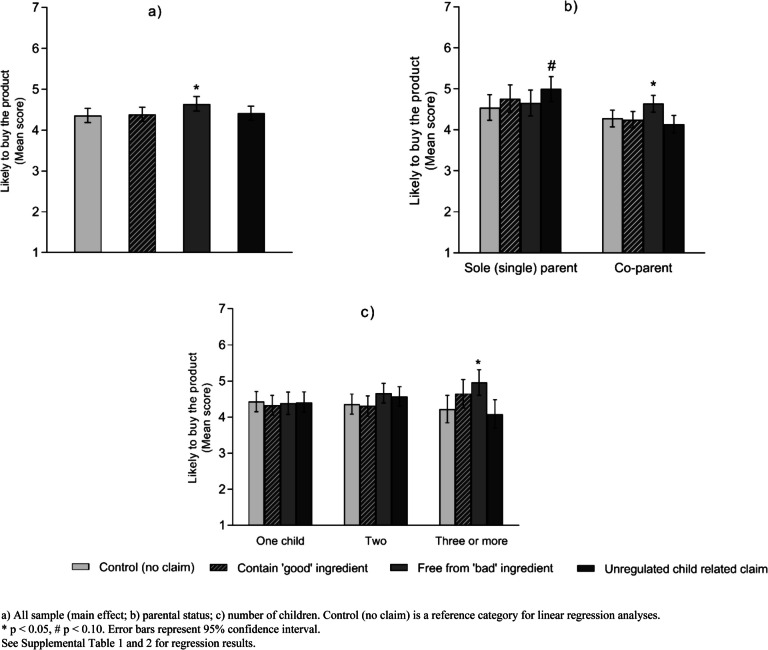
Mean score of participants' purchase intentions for unhealthy toddler food products by claim conditions (*N* = 838)

Compared to the control group, only those who were exposed to ‘free from "bad" ingredient’ claims had significantly higher purchase intentions for unhealthy products (M: 4.36 vs. 4.64; *β* = 0.28, 95%CI 0.04, 0.53; *p* = 0.025) (Fig. 4a and Supplemental Table 1). Two factors modified the effect of claim conditions on product purchase intentions: parental status and number of children in the household (Fig. 4b, c and Supplemental Table 2). Co-parents (M: 4.64 vs. 4.28, *β* = 0.36, 95%CI 0.07, 0.65, *p* = 0.015) and those with three or more children (M: 4.96 vs. 4.22, *β* = 0.74, 95%CI 0.16, 1.31, *p* = 0.012) had higher intentions to purchase unhealthy products when exposed to the free from "bad" ingredient claims compared to those in the control group (Fig. 4 b, c and Supplemental Table 2). Additionally, sole parents (M: 5.00 vs. 4.54, *β* = 0.45, 95%CI -0.00, 0.91, *p* = 0.051) exposed to unregulated 'child-related' claims had moderately higher intentions to purchase the unhealthy products for their toddler compared to control (no claim) (Fig. 4b and Supplemental Table 2).

#### Product perceptions: suitability for toddlers

**Fig. 5:**
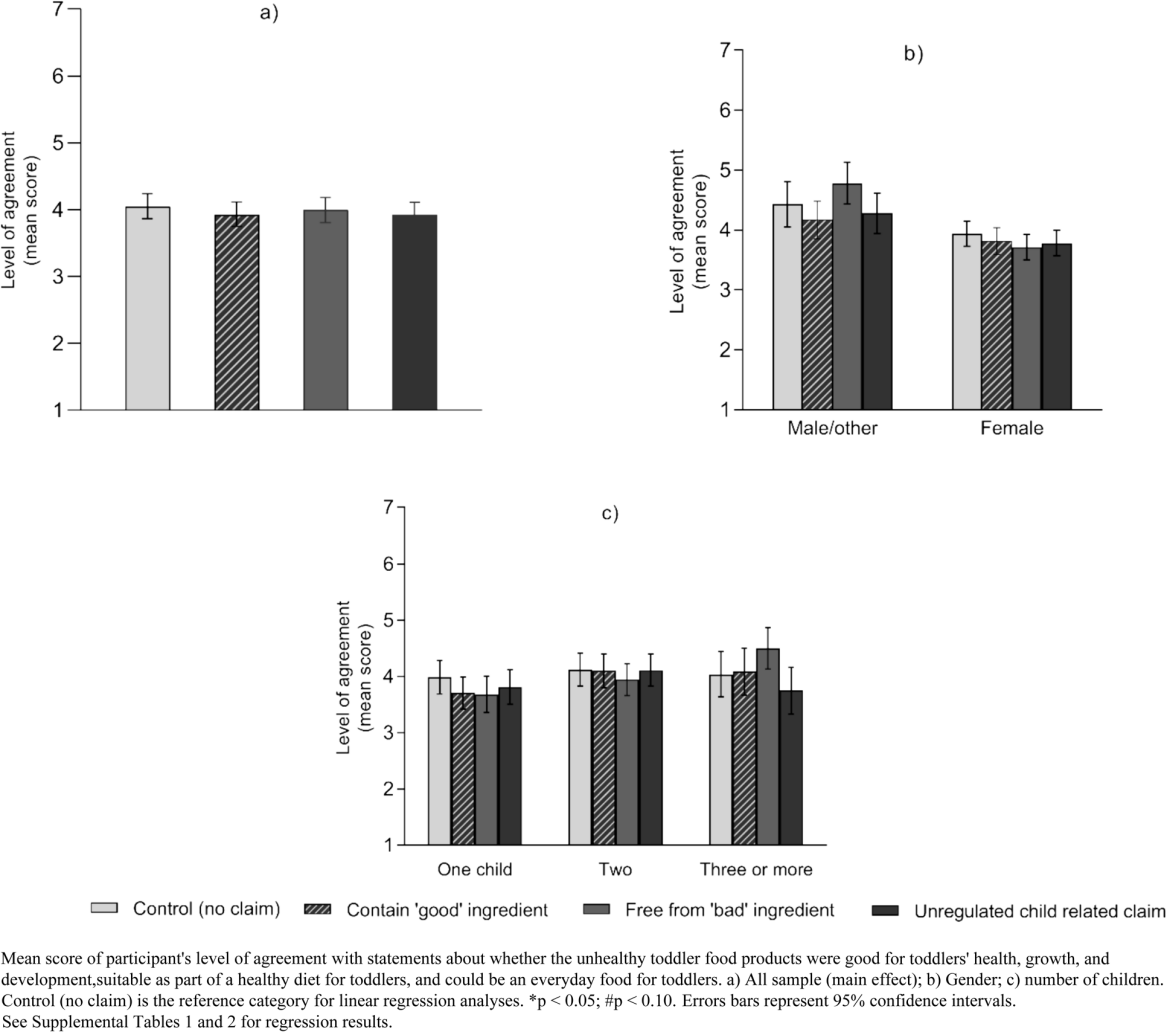
Mean score of participants' perception of unhealthy toddler food products by claim conditions (*N* = 838)

For overall perceptions of the suitability of the products for toddlers, compared to control (no claim), there were no significant differences in perceptions of unhealthy products by claim condition. However, a significant interaction effect was observed between claim condition and participant’s gender (χ2 (3) = 2.33, *p* = 0.0726) and number of children (χ2 (6) = 1.61, *p* = 0.1399) respectively. Specifically, male/other participants and those who had three or more children, who were exposed to ‘free from "bad" ingredient’ claims had higher agreement (non-significant difference) that the products were appropriate for toddlers (Fig. 5 and Supplemental Table 2).

#### Perceived healthiness rating

**Fig. 6:**
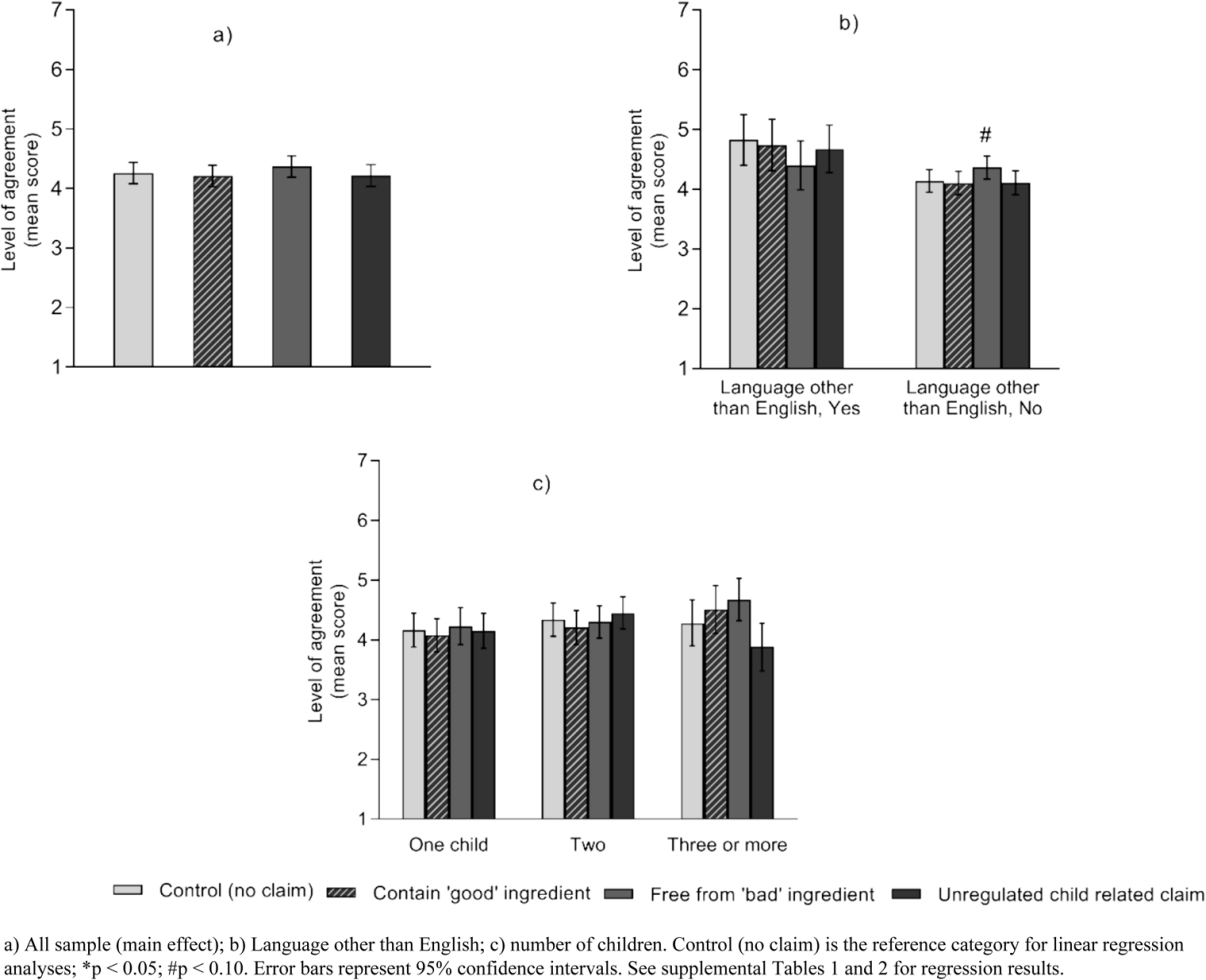
Mean score of participants’ agreement on overall healthiness of the unhealthy products by claim conditions (*N* = 838)

Participants’ mean rating of the perceived healthiness of toddler products did not vary by claim condition (Fig. 6). In addition, there were no subgroup differences by claim conditions except for participants who only speak English. When this subgroup was exposed to ‘free from "bad" ingredient’ claims, they showed higher agreement that the products were healthy compared to control (M: 4.37 vs. 4.14, *β* = 0.23, 95%CI -0.04, 0.49, *p* = 0.098) (Fig. 6 and Supplemental 2).

## Discussion

This online experiment tested whether displaying common claims on unhealthy toddler snack foods influenced parents’ preferences, perceptions, and purchasing intentions for such products. As hypothesised, ‘free from "bad" ingredient’ claims tipped parent’s purchasing intentions towards unhealthy products displaying these claims, with more pronounced effects for certain parent subgroups. These claims also led some demographic subgroups of parents to rate unhealthy products displaying these claims as healthier, perceive them to be more appropriate for toddlers, and to show a greater preference for these products over healthier options, compared to when no claim was displayed. While there was no evidence for the overall sample that ‘contains "good" ingredient’ claims and ‘child-related’ claims enhanced parents’ preferences and purchase intentions for unhealthy toddler snack foods, these two claim types did influence these outcomes for some subgroups of parents. However, there was no evidence these types of claims influenced parent’s views on the healthiness or appropriateness of these products for toddlers. Together, findings indicate that certain claims exert influence on parents’ inclination to buy unhealthy snack foods to feed their young children. As the claims tested here are prevalent on unhealthy infant and toddler food products sold in retail settings [14], the potential reach and impact of such claims on what parents select to purchase to feed their young children is likely to have detrimental effects for young children’s nutrition and diet-related health.

Claims that a product is ‘free from a "bad" ingredient’ (e.g., ‘no additives’, or ‘preservative free’) appear to be most influential, due to their power to promote increased purchasing intentions, and nudge some parent subgroups towards perceiving these products as healthier, more appropriate for toddlers, and leading them to choose these products for their children over healthier alternatives. These findings echo those of McCann et al.’s discrete choice experiment, whereby claims that a toddler snack product is ‘free from a "bad" nutrient’ (regulated claim) or ‘free from a "bad" ingredient’ (unregulated claim) both boosted parents’ perceptions of product healthiness, effectively putting a ‘healthy halo’ on otherwise unhealthy products [10]. It seems that claims stating that an otherwise unhealthy product is free of one negative attribute mislead consumers to assign a more favourable overall evaluation to a product, even though it is high in nutrients of concern such as sugar, salt and saturated fat. As these influential ‘free from "bad" nutrients/ingredients’ claims are the most common types of claims appearing on infant and toddler foods in Australian supermarkets [11, 14, 34], their consumer reach and impact is likely to be widespread.

On-pack claims stating that a toddler snack food ‘contains a "good" ingredient’ (e.g., ‘contains real vegetables’) did not influence all parents’ product preferences, purchase intentions or perceptions of unhealthy toddler foods. However, these claims did influence the sub-group of parents with three or more children to show a greater preference for unhealthy toddler snack foods displaying these claims. Thus, our findings are partially consistent with previous studies conducted with parents of young children in different populations and for different products. For example, several studies with United States (US) parents found that similar, nutrition-related claims (e.g., 100% all natural) led parents to believe sugary drinks and cereals were healthier and encouraged preference for such products over healthier alternatives [35, 36]. Similarly, on-pack claims such as ‘high in fibre’ and ‘contains whole grains’ influenced Chilean adults to perceive unhealthy cereals more favourably and increased intentions to purchase and recommend these products [20]. The lack of effect of ‘contains "good" ingredient’ claims on parents’ purchase intentions and perceptions of toddler snack foods in our study is not clear. It could be that most parents disregarded these claims as mere marketing, or that some aspect of our study methodology was not sensitive to detect effects of these claims (see limitations). Nonetheless, our overarching finding that claims that a product is ‘free from a "bad" ingredient’ were more influential than claims that a product ‘contains a "good" ingredient’ aligns with evidence from other studies which have found that claims with *risk-avoidance* appeals are more appealing and persuasive than *benefit-seeking* claims in influencing consumer’s product perceptions [21, 26].

Our study also tested parent’s responses to unregulated ‘child-related’ or developmental claims (e.g., ‘right texture to encourage chewing’, ‘encourages self-feeding’, ‘perfect for little hands’). In general, results indicated that such claims did not significantly affect parents’ product choice, purchasing intentions or perceptions, with the exception that sole parents who viewed unhealthy products displaying these claims showed higher purchase intentions for these products. Despite the common practice of displaying 'child-related' claims on toddler foods in Australia and elsewhere [37, 38], few published studies appear to have assessed effects of such claims on consumers [10, 39, 40]. Further studies are needed to expand our understanding of how 'child-related' claims impact parent’s perceptions, preferences, and intentions to purchase these foods for their children.

A study with US parents’ found that neither the demographic characteristics of the parent, or their child, moderated the impact of nutrition-related claims on drink selection, suggesting these claims were equally impactful with all parents [22]. However, our moderation analyses revealed that some demographic subgroups of Australian parents were more susceptible to influence by the claims tested than others. ‘Free from "bad" ingredient’ claims were particularly influential with co-parents and parents with three or more children, prompting them to choose a higher number of unhealthy toddler snack foods and show stronger intentions to buy these products for their toddlers. ‘Free from "bad" ingredient’ claims also enhanced perceptions of the appropriateness of these products for toddlers among parents with three or more children and male/non-binary parents. Additionally, parents with three or more children were influenced by ‘contains "good" ingredient’ claims to choose unhealthy products more often. The finding that parents with larger families were more vulnerable to influence by both types of ingredient-related claims is especially concerning as parents with larger families are responsible for shopping for and feeding a greater number of children. The busy schedules of parents caring for multiple children may also lead them to rely more on ready-made toddler foods for convenience rather than preparing home-cooked meals [15, 41]. Interestingly, sole parents, who may also be especially time-poor, showed stronger intentions to buy unhealthy toddler snack foods when they displayed ‘child-related’ claims, suggesting these claims appealed to this parent subgroup. Unexpectedly, parents with a language other than English chose fewer unhealthy products when exposed to ‘child-related’ claims, suggesting these claims backfired as a promotional strategy with this subgroup. Further research testing additional beliefs and behaviours surrounding household food purchasing and preparation could help unravel our observed moderating effects of demographic characteristics on responses to certain types of claims. Overall, it seems that some types of claims are an effective promotional tactic with some parent target audiences and not others.

### Strengths & limitations

Conducting a controlled experiment and testing the appearance of claims on professionally designed, mock products should have helped minimise bias and confounding factors, since participants held no prior knowledge or brand attitudes towards the products tested. Our sample comprised parents/carers of toddlers who varied in demographic characteristics, enabling us to assess whether certain sub-groups of parents are more influenced by claims than others. We also assessed a wider range of responses to the claims than some previous studies, that have assessed a single outcome, such as perceived healthfulness.

Limitations should be also noted. Firstly, brief exposure to claims in an online environment may have been insufficient to yield strong evidence of the impact of claims across all parents on all the response measures. Future research employing designs with stronger ecological validity (e.g., assessing parents' purchasing behaviour in response to claims appearing on real toddler food products) would expand our understanding of how these claims operate. Secondly, this study assessed single claims on toddler foods and this may not have as strong an impact as when multiple claims are displayed on a single product, as is common in Australia, with some infant and toddler foods found to display up to 26 claims [11]. Future research could overcome these limitations by testing the impact of multiple claims and repeated exposure to claims on parent’s product perceptions, preferences and purchase intentions. Third, social desirability bias may have led participants to select healthier product options than they would have otherwise, but this potential influence is likely to have been consistent across all randomised subgroups.

The use of *p* < 0.20 for interaction analysis may increase the risk of Type I errors, potentially leading to false positives, so these results should be interpreted with caution. Conversely, employing a low alpha level (*p* < 0.05) for the interaction term can result in false negatives, particularly in studies involving complex human behaviours and interactions. Increasing the alpha level or error rate to 20% to detect potential interactions can enhance test power. Therefore, it is crucial to use a higher Type I error rate when assessing interactions, as previously suggested. However, careful consideration of the trade-off between the increased likelihood of detecting true effects and the risk of Type I errors is essential when interpreting findings.

Our sample of Australian parents of toddlers was recruited from an online panel, rather than being drawn from the general population, and while our sample achieved a spread of demographic characteristics, it is unclear to what extent our findings are generalisable to the broader Australian population. As this online experiment was conducted in an artificial setting with relatively brief exposure to claims, findings may not necessarily generalise to actual purchasing preferences in ‘real world’ food retail settings. A previous US study that used a virtual convenience store to assess effects of claims found stronger evidence of influence for nutrition-related claims than we detected in our study [36].

### Implications for policy/practice

Together with prior research, findings from this study will help inform policy decisions and evidence-based advocacy regarding the regulation of claims on packaged toddler foods. While the present study did not find as clear effects of claims on parent’s product perceptions and preferences as some previous studies, the effects observed generally indicated that claims enhanced the appeal of unhealthy toddler snack food products. Furthermore, the no claim condition was least conducive to parents favouring unhealthy products over healthier alternatives, suggesting the best way to eliminate potential detrimental effects of claims on consumer’s food choices would be to ban such claims on unhealthy products altogether. Setting nutritional thresholds, such as those proposed by WHO Europe’s NPPM [18] and prohibiting infant and toddler food products from displaying such claims would enable parents to more accurately evaluate the healthfulness of products, and reorient their preferences and purchases towards healthier options. Additionally, Government could require that warning labels be displayed on products high in nutrients of concern, as warning labels have been found to mitigate but not eliminate ‘health halo’ effects of nutrition-related content claims on product perceptions and preferences [20, 26, 42]. More specifically, studies with US parents have found that displaying added sugar warning labels on sugary snacks and drinks encourages parents to choose healthier options for their children [39, 43]. Overall, available evidence suggests that stricter regulation surrounding the marketing and labelling of toddler foods needs to be introduced to protect parents against potentially misleading claims and encourage healthier choices. Ultimately, this could promote population-level improvements in the foods consumed by toddlers, improving their dental health, and reducing their longer-term risk of obesity and diet-related chronic disease.

Additionally, our finding that certain subgroups of parents may be especially vulnerable to being misled by marketing claims on toddler foods suggests that until stronger restrictions on nutritional composition and claims on unhealthy toddler foods are implemented, targeted educational interventions are needed to increase parents’ awareness of the suboptimal nutritional profile of many ready-made infant and toddler foods, and to enhance their ability to critically evaluate claims and product ingredients to identify the healthiest options for their young children. More stringent regulation of claims alone may not be a sufficient approach to address the disproportionately impacted parents, as other underlying causes may be at play. Some parents may need additional support to make informed and healthy food choices for their children and to ensure regulations have equitable impacts.

### Conclusions

This study adds a valuable contribution to understanding how various on-pack claims influence the product preferences, purchase intentions and perceptions of parents of toddlers aged 12 to < 36 months. Findings suggest that certain claims, especially the most common types of claims, that a product is ‘free from "bad" ingredients’, increase parents’ preferences and purchase intentions for unhealthy toddler snack foods. Some demographic subgroups, including co-parents and parents with three or more children may be more influenced by these claims. This study found weaker evidence that claims a product ‘contains a "good" ingredient’ and unregulated ‘child-related’ claims influence parents’ preferences, intentions, and perceptions towards unhealthy toddler foods. However, considering mounting evidence from other studies, we recommend such claims should not be permitted on infant and toddler foods, for their possible role in promoting inaccurate product perceptions and unhealthy product choices by parents for their young children.

### Supplementary Information

Supplementary Material 1.

## Data Availability

The datasets used and/or analysed during the current study are available from the corresponding author on reasonable request.

## Abbreviations

ADGs: Australian Dietary Guidelines
UP: Ultra-processed
UPFs: Ultra-processed foods
FSANZ: Food Standards Australia New Zealand
FSC: Food Standard Code
mg: Milligram
g: Gram
NPPM: Nutrient promotion and profile model
WHO: WORLD Health Organization
NIP: Nutrition information panel
SES: Socio-economic status
SEIFA: Socio-economic index for areas
US: United States
