# Supplementary material for: Effects of marketing claims on toddler food products on parents’ product preferences, perceptions and purchasing intentions: an online experiment
Source: Int J Behav Nutr Phys Act. 2024 May 21;21:60. doi: 10.1186/s12966-024-01603-9 (PMC11110258; doi:10.1186/s12966-024-01603-9)
Supplement: Supplementary file 1 — Supplementary Material 1. [file 12966_2024_1603_MOESM1_ESM.docx]

**Supplemental File:**

**Supplemental Table 1: Regression models testing effects of marketing claim condition on parents’ responses on toddler food products (*n*=838)**

|  | Marketing claim condition | | | | | | | | | | | |
| --- | --- | --- | --- | --- | --- | --- | --- | --- | --- | --- | --- | --- |
|  | Control (no claim)  (*n*=209) | | | Contains ‘good ingredient.  (*n*=209) | | | Free from ‘bad’ ingredient  (*n*=210) | | | Unregulated child related claim  (*n*=210) | | |
| Outcome | *Mean* | *β* | *p* | *Mean* | *β* | *p* | *Mean* | *β* | *p* | *Mean* | *β* | *p* |
| Product choice (number of times parents chose an unhealthy products) | 1.64 | *ref* | | 1.68 | 0.03 | 0.650 | 1.74 | 0.06 | 0.293 | 1.58 | -0.04 | 0.524 |
| Product purchase intentions | 4.36 | *ref* | | 4.38 | 0.03 | 0.836 | 4.64 | 0.28 | **0.025** | 4.42 | 0.06 | 0.648 |
| Product perception (appropriate for toddlers) | 4.05 | *ref* | | 3.93 | -0.12 | 0.374 | 4.00 | -0.06 | 0.671 | 3.93 | -0.13 | 0.348 |
| Product Healthiness rating | 4.26 | *ref* | | 4.21 | -0.05 | 0.710 | 4.37 | 0.11 | 0.378 | 4.22 | -0.04 | 0.780 |

**Notes:** *β* = regression coefficient; *ref* = referent category in Poisson/linear regression model. For the product choice task, the number of times parents chose an unhealthy product from each of the four product pairs was counted (range: 0-4). For purchase intentions, parents were required to rate their likelihood of purchasing that product for their toddler on a 7- point scale ranging from ‘very unlikely’ to ‘very likely’ and a composite mean score (1-7) of these ratings was computed to assess overall intentions for purchasing unhealthy toddler snack food products. Participants level of agreement (ranging from ‘strongly disagree’ =1 to ‘strongly agree=7’) with statements about whether the unhealthy toddler food products were: - *good for toddlers’ health, growth, and development, suitable as part of a healthy diet for toddlers, could be an everyday food for toddlers.* and the level of agreement with each statement was combined to obtain composite mean score (1-7), where higher scores indicated greater agreement, these products were appropriate for toddlers. Poisson regression (count variable: number of times choosing unhealthy products) and linear regression (continuous outcomes) were used. Numbers in bold are significant at *p*<0.05.

**Supplemental Table 2: Regression models for socio-demographic that shown an interaction effect with marketing claim condition (Subgroup analysis) (*n*=838)**

| Outcome | Subgroup | Marketing claim condition | | | | | | | | | | |
| --- | --- | --- | --- | --- | --- | --- | --- | --- | --- | --- | --- | --- |
|  |  | Control (no claim)  (*n*=209) | | Contains ‘good ingredient  (*n*=209) | | | Free from ‘bad’ ingredient  (*n*=210) | | | Unregulated child related claim  (*n*=210) | | |
|  |  | *Mean* | *β / p* | *Mean* | *β* | *p* | *Mean* | *β* | *p* | *Mean* | *β* | *p* |
| Product choice (number of times parents chose an unhealthy products) | **Parental status** |  |  |  |  |  |  |  |  |  |  |  |
|  | Sole/sing parent | 1.88 | *ref* | 1.73 | -0.08 | 0.415 | 1.69 | -0.11 | 0.257 | 1.75 | -0.07 | 0.414 |
|  | Co-parent | 1.54 | *ref* | 1.67 | 0.08 | 0.241 | 1.77 | 0.14 | **0.053** | 1.50 | -0.02 | 0.766 |
|  | **Language other than English** |  |  |  |  |  |  |  |  |  |  |  |
|  | Yes | 1.97 | *ref* | 1.86 | -0.06 | 0.603 | 1.72 | -0.14 | 0.259 | 1.52 | -0.26 | **0.015** |
|  | No | 1.57 | *ref* | 1.65 | 0.05 | 0.459 | 1.75 | 0.11 | **0.099** | 1.60 | 0.01 | 0.831 |
|  | **Number of children** |  |  |  |  |  |  |  |  |  |  |  |
|  | One child | 1.70 | *ref* | 1.61 | -0.05 | 0.560 | 1.56 | -0.09 | 0.384 | 1.54 | -0.10 | 0.316 |
|  | Two | 1.70 | *ref* | 1.73 | 0.01 | 0.879 | 1.72 | 0.01 | 0.895 | 1.60 | -0.06 | 0.463 |
|  | Three and above | 1.41 | *ref* | 1.76 | 0.22 | 0.081 | 2.02 | 0.36 | **0.001** | 1.62 | 0.14 | 0.281 |
| Product purchase intentions |  |  |  |  |  |  |  |  |  |  |  |  |
|  | **Parental status** |  |  |  |  |  |  |  |  |  |  |  |
|  | Sole/sing parent | 4.54 | *ref* | 4.76 | 0.22 | 0.364 | 4.66 | 0.11 | 0.628 | 5.00 | 0.45 | **0.051** |
|  | Co-parent | 4.28 | *ref* | 4.25 | -0.03 | 0.851 | 4.64 | 0.36 | **0.015** | 4.14 | -0.13 | 0.373 |
|  | **Number of children** |  |  |  |  |  |  |  |  |  |  |  |
|  | One child | 4.43 | *ref* | 4.33 | -0.10 | 0.622 | 4.38 | -0.05 | 0.810 | 4.41 | -0.02 | 0.939 |
|  | Two | 4.36 | *ref* | 4.31 | -0.05 | 0.783 | 4.66 | 0.30 | 0.116 | 4.57 | 0.21 | 0.264 |
|  | Three and above | 4.22 | *ref* | 4.65 | 0.42 | 0.168 | 4.96 | 0.74 | **0.012** | 4.08 | -0.14 | 0.651 |
|  | **Gender** |  |  |  |  |  |  |  |  |  |  |  |
|  | Male/Other | 4.43 | *ref* | 4.17 | -0.26 | 0.308 | 4.78 | 0.35 | 0.203 | 4.28 | -0.15 | 0.569 |
|  | Female | 3.94 | *ref* | 3.82 | -0.12 | 0.430 | 3.71 | -0.23 | 0.133 | 3.78 | -0.15 | 0.307 |
|  | **Number of children** |  |  |  |  |  |  |  |  |  |  |  |
|  | One child | 3.99 | *ref* | 3.71 | -0.28 | 0.159 | 3.68 | -0.31 | 0.146 | 3.81 | -0.18 | 0.387 |
|  | Two | 4.12 | *ref* | 4.10 | -0.02 | 0.928 | 3.95 | -0.18 | 0.385 | 4.11 | -0.01 | 0.961 |
|  | Three and above | 4.04 | *ref* | 4.09 | 0.04 | 0.896 | 4.50 | 0.46 | 0.150 | 3.75 | -0.30 | 0.375 |
| Product Healthiness rating | **Language other than English** |  |  |  |  |  |  |  |  |  |  |  |
|  | Yes | 4.83 | *ref* | 4.74 | -0.08 | 0.809 | 4.40 | -0.43 | 0.204 | 4.67 | -0.15 | 0.642 |
|  | No | 4.14 | *ref* | 4.10 | -0.04 | 0.787 | 4.37 | 0.23 | **0.098** | 4.11 | -0.03 | 0.826 |
|  | **Number of children** |  |  |  |  |  |  |  |  |  |  |  |
|  | One child | 4.16 | *ref* | 4.08 | -0.09 | 0.660 | 4.23 | 0.07 | 0.747 | 4.15 | -0.02 | 0.937 |
|  | Two | 4.34 | *ref* | 4.21 | -0.13 | 0.520 | 4.30 | -0.03 | 0.864 | 4.45 | 0.11 | 0.572 |
|  | Three and above | 4.28 | *ref* | 4.51 | 0.22 | 0.461 | 4.67 | 0.39 | 0.172 | 3.88 | -0.40 | 0.179 |

**Notes:** *β* = regression coefficient; *ref* = referent category in Poisson/linear regression model. For the product choice task, the number of times parents chose an unhealthy product from each of the four product pairs was counted (range: 0-4) and Poisson regression was used. For purchase intentions, parents were required to rate their likelihood of purchasing that product for their toddler on a 7- point scale ranging from ‘very unlikely’ to ‘very likely’ and a composite mean score (1-7) of these ratings was computed to assess overall intentions for purchasing unhealthy toddler snack food products. Participants level of agreement (ranging from ‘strongly disagree’ =1 to ‘strongly agree=7’) with statements about whether the unhealthy toddler food products were: - *good for toddlers’ health, growth, and development, suitable as part of a healthy diet for toddlers, could be an everyday food for toddlers.* and the level of agreement with each statement was combined to obtain composite mean score (1-7), where higher scores indicated greater agreement, these products were appropriate for toddlers. Linear regression analyses were performed for these continuous outcomes. Numbers in bold are significant at *p*<0.1.
